# Sex, bugs and Haldane's rule: the nematode genus *Pristionchus *in the United States

**DOI:** 10.1186/1742-9994-3-14

**Published:** 2006-09-12

**Authors:** Matthias Herrmann, Werner E Mayer, Ralf J Sommer

**Affiliations:** 1 Max Planck Institute for Developmental Biology, Department of Evolutionary Biology, Tübingen, Germany

## Abstract

**Background:**

The nematode *Pristionchus pacificus *has been developed as a satellite organism in evolutionary developmental biology for comparison to *Caenorhabditis elegans*. Comparative studies have revealed major differences in the regulation of developmental processes between *P. pacificus *and *C. elegans*. To place evolutionary developmental biology and the observed developmental differences between species in a comprehensive evolutionary context, such studies have to be complemented with ecological aspects. Knowledge about the ecology of the organism in question might indicate specific environmental conditions that can result in developmental adaptations and could account for species differences in development. To this end, we have started to investigate the ecology of *Pristionchus *nematodes. In recent field studies in Western Europe we found six *Pristionchus *species that are closely associated with scarab beetles and the Colorado potato beetle. This *Pristionchus *– beetle association provides the unique opportunity to combine research in evolutionary developmental biology with ecology. However, it remains unknown how general these findings from Europe are on a global scale.

**Results:**

Here, we describe the *Pristionchus *species associated with scarab and Colorado potato beetles in the Eastern United States and show striking transatlantic differences and unexpected evolutionary and ecological patterns. Twohundredeighty of 285 (98%) isolates from American scarab beetles belong to five *Pristionchus *species, all of which are different from the European species. We describe four of them as novel *Pristionchus *species. The five American *Pristionchus *species fall into a single phylogenetic clade and have a male-female (gonochoristic) mode of reproduction, whereas the majority of European isolates are hermaphroditic. Crosses between the two most closely related species, *P. aerivorus *and *P. pseudaerivorus *n. sp., follow Haldane's rule in that heterogametic F1 males are inviable. We observed *P. aerivorus *and *P. pseudaerivorus *n. sp. coexisting on the same scarab beetle and obtained two cases of F1 hybrids from wild beetles. Finally, the Colorado potato beetle is associated with the same nematode, *P. uniformis *in the United States and Europe. Given the introduction of the Colorado potato beetle to Europe in 1877, our results suggest that *P. uniformis *was introduced together with its beetle vector.

**Conclusion:**

Taken together, the *Pristionchus *– beetle association provides a powerful tool for studying biodiversity, biogeography, speciation and species invasion on a global scale.

## Background

The diplogastrid nematode *Pristionchus pacificus *has been developed as a satellite organism in evolutionary developmental biology [1]. *P. pacificus *is a hermaphroditic species that can feed on *E. coli *and has a 3–4 day generation time (20°C) [2]. Original studies in *P. pacificus *concentrated on the developmental, genetic and molecular analysis of sex determination, vulva and gonad formation [3]. More recently, a genomic initiative including the generation of a genetic linkage map and a physical map has complemented the developmental and genetic studies [4,5]. A whole genome sequencing project is currently ongoing and should result in a draft of the complete genome sequence in the near future [1].

In general, little is known about the ecology of the nematode species used as laboratory organisms, such as *P. pacificus *and *C. elegans*. For example, the environmental niche of the model organism *C. elegans *is largely unknown and only very recently did several studies indicate that *C. elegans *occurs predominantly in compost heaps [6,7]. We have recently shown that nematodes of the genus *Pristionchus *live in close association with scarab beetles and the Colorado potato beetle (CPB) in Western Europe [8]. Intensive samplings in 2004 and 2005 generated 371 isolates that fell into six species, most of which are morphologically indistinguishable from one another. The two hermaphroditic species *P. entomophagus *and *P. maupasi *accounted for 226 of these 371 (60%) isolates and occurred on dung beetles and cockchafers, respectively. However, the satellite organism *P. pacificus *was neither observed on scarab beetles nor on the CPB in Western Europe.

In total, 27 species of the genus *Pristionchus *are described in the literature, but only four of these descriptions are younger than 1958 and many are from the 19th century [9]. Also, many of these descriptions are rather short and lack illustrations. Type material does not exist in most cases – either because it never existed or because it got lost. Furthermore, it has long been known that morphometric values of *Pristionchus *species change during culture under laboratory conditions [10]. Taking this high phenotypic plasticity into account, the 27 species descriptions contain most likely a number of synonyms. Facing all of these problems of „classical“ taxonomy, we have started to use a novel methodology to investigate the biodiversity and phylogeny of *Pristionchus *nematodes. Following the isolation of *Pristionchus *nematodes from wild caught beetles, we establish isogenic female lines. These isogenic female lines are first processed by *SSU *sequence analysis for molecular barcoding. Previous studies have indicated that this method is robust and provides a clear indication for species identity or evidence for the existence of a novel species [8]. To confirm the species identification by molecular barcoding we perform mating experiments of a new isolate with the reference strain of the representative species. For the reference strain of a novel species, morphometric measurements are provided in addition to the molecular sequence tag. Also, a frozen stock collection has been established, type material is delivered to museums and strains are available upon request. We strongly believe that this comprehensive methodology – molecular barcoding, mating experiments, morphometric measurements, frozen stock collection and type material at museum collections – provides the optimal tool for species identification and analysis in this group of nematodes.

In the present study we surveyed the association of *Pristionchus *species with scarab beetles and the CPB in the Eastern United States, particularly in the states of New York, Massachusetts, Nebraska, Ohio, Iowa, Texas, and Kansas. The two studies indicate striking differences in the species composition, mode of reproduction and speciation frequency of *Pristionchus *species on the two continents. More than 98% of the *Pristionchus *isolates from the Eastern United States represent five species, all of which are unknown from Europe. Given the rationale described above, we describe four novel *Pristionchus *species. These results establish *Pristionchus *as a nematode model system for biogeography and biodiversity and represent an important difference to the biogeography of *Caenorhabditis *species. In addition, the analysis of *Pristionchus *species in the US provided two unexpected cases that help to establish *Pristionchus *as a future model system in two additional areas of evolutionary biology and ecology, namely speciation and species invasion. Crosses between the two most closely related American species, *Pristionchus aerivorus *and *Pristionchus pseudaerivorus *n. sp., result in inviable males following Haldane's rule. Haldane's rule states that in hybrids between diverging species the sterile, absent or underrepresented offspring is of the heterogametic sex [11]. Finally, the CPB is associated with the same nematode, *Pristionchus uniformis *in the US and Europe. Given the introduction of the CPB to Europe in 1877, these results suggest that *P. uniformis *was introduced together with its beetle vector. Species invasion is a common phenomenon in contemporary ecology and biogeography and the *P. uniformis *case reported in this study might represent a useful tool for future genetic and molecular studies on species invasion.

## Results

### Five of the *Pristionchus *species found on scarab beetles in the US do not occur in Europe

**Figure 1:**
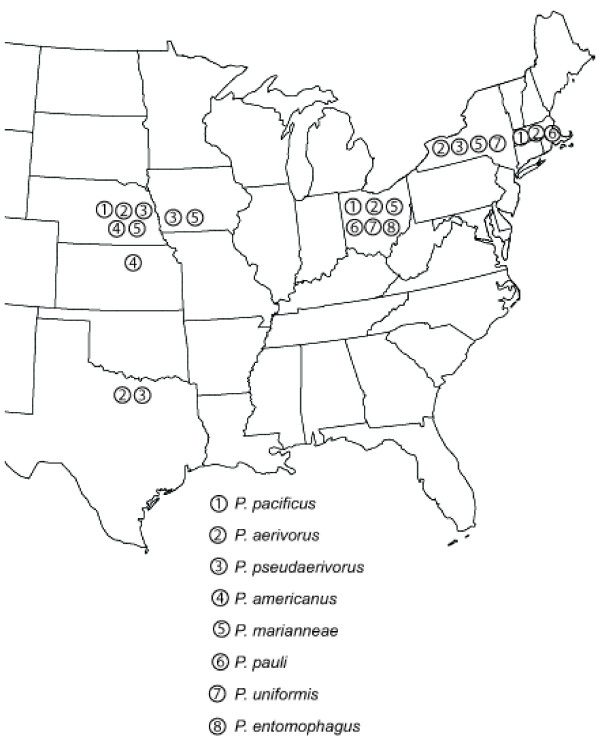
**Map of the Eastern US showing the distribution of sampled *Pristionchus *species**. Material has been obtained from the following sampling sites: Massachusetts (Two locations: Carver and Centerville), New York (six locations: Bellona, Waterloo, Hoover farm, golf court I and II, Geneva), Ohio (One location : Wooster), Iowa (One location: near Bellevue, Nebraska), Kansas (One location: Greensburg), Texas (One location: Austin), and Nebraska (five locations: Lincoln: pioneer park, wilderness park, nine mile prairie; Bennett, Nemaha).

**Figure 2:**
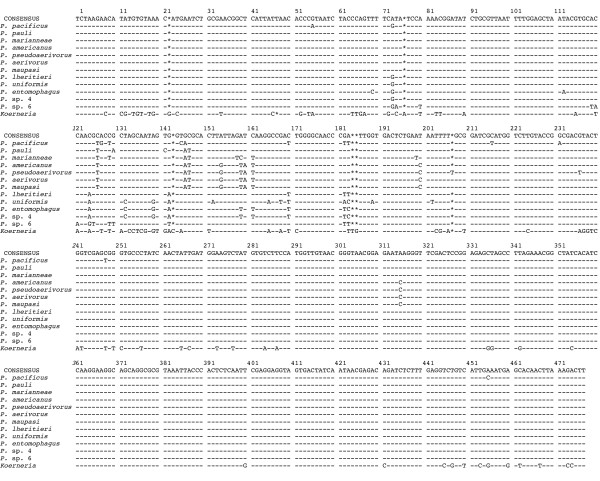
**Alignment of *SSU *sequences**. Sequences of 13 *Pristionchus *and one *Koerneria *species were aligned manually. Dashes (-) indicate identity to the majority consensus sequence at the top; asterisks (*) indicate alignment gaps. Numbering refers to the *SSU *segment obtained in this study. The sequences have been submitted to the GenBank database and are available under the accession codes DQ270018–DQ270025 and DQ419900–DQ419904.

To determine if *P. pacificus *and other species of the genus *Pristionchus *are associated with scarab beetles in the United States, we analyzed beetles from Massachusetts, New York, Ohio, Iowa, Kansas, Texas, and Nebraska. The scarab beetle fauna differs between North America and Europe. For example, the cockchafer *Melolontha melolontha *and the dung beetle *Geotrupes stercorosus *that host the predominant European *Pristionchus *species are not known from North America. However, the subfamily Melolonthinae, which includes the june beetles and chafers, contains more than 500 species in North America, some of which are serious pests [12]. We used the same sampling strategies in Europe and the United States and collected scarab beetles from the US that are comparable with those obtained in our European study. However, we have not been able to sample identical species on both continents. In total, we investigated 1241 beetles of more than 15 genera yielding 285 *Pristionchus *isolates (Fig. 1, Table 1). *SSU *sequence analysis and mating experiments of the 285 isolates revealed that they fall into seven species, two of which were known to us (Fig. 2). We obtained two strains of *P. entomophagus *(both from beetles from Ohio), a hermaphroditic species that is one of the most common *Pristionchus *species in Europe [8]. We also found *P. pacificus*, but in no more than three beetle samples, one from Ohio, one from Nebraska and one from Massachusetts. Surprisingly, the remaining 280 (98%) isolates represent five *Pristionchus *species, which had up to then neither been found by beetle sampling in Western Europe [8] nor by previous soil sampling in the United States [13].

**Table 1:** Frequencies of *Pristionchus *isolates on beetles.

Beetle species	No. of individuals		*Pristionchus *species						
		*P. aerivorus*	*P. pseudaerivorus*	*P. marianneae*	*P. pauli*	*P. uniformis*	*P. pacificus*	*P. entomophagus*	*P. americanus*
	
Scarabaeidae:									
*Phyllophaga *spp.	621	57 (9.2%)	46 (7.4%)	25 (4%)	1(0.2%)	-	1 (0.2%)	2 (0.4%)	1(0.2%)
*Popilia japonica*	136	15 (11%)	1 (0.7%)	18 (13.2%)	1 (0.7%)	-	-	-	-
*Cyclocephala lurida*	49	5 (10.2)	23 (46.9%)	-	-	-	1 (2%)	-	-
*A. solstitiale*	8	3 (37.5%)	-	1 (12.5%)	-	-	-	-	-
*Polyphylla variolosa*	6	-	-	-	2 (33%)	-	-	-	2(33%)
*Maladera castanea*	62	3 (4.8%)	-	-	-	-	-	-	-
*Diplotaxis *spp.	25	-	1 (4%)	-	-	-	-	-	-
*Serica sericea*	74	-	-	1 (1.4%)	-	-	-	-	-
*Lichnanthe vulpina*	60	-	-	-	22 (36.7%)	-	-	-	-
*Hoplia equina*	11	-	-	-	9 (81.9%)	-	-	-	-
*Anomala orientalis*	50	-	-	-	-	-	1 (2%)	-	-
*Aphodius *spp.	82	-	-	-	-	-	-	-	-
*Onthophagus *spp.	6	-	-	-	-	-	-	-	-
*Ateuchus histeroides*	6	-	-	-	-	-	-	-	-
Chrysomelidae:									
*L. decemlineata*	110	-	-	-	-	42 (38.2%)	-	-	-
Hydrophilidae:									
*Hydrophilus *spp.	39	-	-	-	-	-	-	-	-

Nematode species of the genus *Pristionchus *have little diagnostic morphological characters and usually show overlapping ranges of morphometric values [14,8]. Prior to our own studies on *P. pacificus*, only one *Pristionchus *species, *P. aerivorus*, was described for North America by Cobb in 1916 [9,15]. All previously obtained strains of *P. pacificus *from North America result from soil samples taken by various researchers [8,13]. It is important to note that all of these soil-derived *P. pacificus *strains represent random findings within a sampled locality. *P. pacificus *was a rare nematode in these soil samples and multiple isolates from the same soil sample are not available.

Morphometric analysis revealed that the most common species in our analysis is very similar to *P. aerivorus *(Table 2). Thus, we have classified this species as *P. aerivorus*. The four other species are described as novel species and we provide morphological measurements, molecular *SSU *sequence data, the results of mating experiments and species designations for all of them (Fig. 2, Table 2, see species diagnosis below). The reference strains of all species are available as living cultures and frozen stocks in our laboratory and can be provided to other researchers upon request. Also, type material has been delivered to museums. Together, these five species account for the majority of isolates from scarab beetles (Table 1). Specifically, we obtained *P. aerivorus *(85 isolates), *P. pseudaerivorus *n. sp. (71 isolates), *P. marianneae *n. sp. (45 isolates), *P. pauli *n. sp. (35 isolates) and *P. americanus *n. sp. (1 isolate) in the US scarab beetle survey. In the following, we provide the diagnoses for the four novel *Pristionchus *species:

**Table 2:** Morphometric measurements of the *Pristionchus *species

	*P. aerivorus *(Cobb in Meirill & Ford 1916)		*P. aerivorus *RS 5106		*P. pseudaerivorus *RS 5139		*P. marianneae *RS 5108		*P. paulii *RS 5130		*P. americanus *RS 5140	
	females	males	females	males	females	males	females	males	females	males	females	males
L	1500	800	1494 (1265–1683)	992 (868–1101)	1715 (1496–1934)	1128 (850–1333)	1277 (983–1496)	878 (761–983)	1437 (1077–1809)	1018 (972–1069)	1207 (882–1510)	1103 (938–1392)
L'	1305	712	1281	863	1542	1028	1113	795	1240	908	1042	989
			(1045–1464)	(746–943)	(1306–1731)	(779–1212)	(836–1290)	(689–896)	(898–1619)	(851–950)	(749–1329)	(820–1282)
mbd	88.5	80	97(71–122)	68(51–80)	139(119–158)	97(72–114)	83(67–99)	54(51–57)	94(77–121)	64(53–69)	118(92–141)	92(73–125)
sl	12	7,2	7(4.9–9.3)	5.6(4.2–7.6)	7.9(6.9–9.3)	6.7(5.4–7.8)	8.4(7.6–9.6)	7.5(6.1–10.2)	9.6(7.8–11.5)	8.9(7.0–10.1)	11.6(10.1–14.7)	8.1(6.1–11)
sd			8(7–10)	6(4–9)	8(7–9)	8(6–8)	8(7–10)	7(6–7)	7(6–9)	7(7–8)	9.3(6.9–12.8)	8.2(6.8–10.4)
Pl	180	120	176(168–184)	153(144–159)	193(183–198)	174(160–188)	176(157–189)	154(142–163)	208(184–229)	184(172–191)	181(163–199)	176(160–190)
cl	133.5	88	106(98–112)	92(83–97)	113(106–118)	101(89–112)	108(99–115)	91(80–96)	138(119–147)	116(111–122)	112(101–121)	107(90–115)
c/p	73.8	73.3	60(58–62)	60(52–63)	60(58–61)	58(55–60)	61(59–63)	59(56–62)	67(62–77)	63(61–66)	62(59–64)	61(57–64)
mbbd			33(29–36)	23(20–26)	32(29–36)	26(22–29)	33(28–36)	25(23–27)	33(27–41)	27(25–30)	30(27–33)	24(21–28)
tbd			31(27–35)	23(21–26)	36(30–44)	29(23–32)	28(24–34)	22(20–24)	33(25–43)	27(23–30)	32(28–38)	28(24–30)
ae-v	765		667(592–763)		837(754–883)		639(473–751)		731(583–885)		605(379–788)	
rl			51(47–58)	44(41–48)	45(35–56)	44(35–48)	43(37–49)	38(35–42)	40(33–46)	36(31–44)	43(31–53)	43(33–54)
abd	39	36.8	39(34–48)	36(33–39)	49(44–53)	51(44–58)	38(33–41)	40(39–41)	40(32–49)	42(37–47)	46(38–55)	46(35–59)
spic		55.2		57(52–62)		60(50–68)		52(46–55)		50(40–58)		50(46–57)
gub		18.4		25(24–26)		25(23–29)		25(23–30)		24(17–29)		22(20–24)

Legend: L: total length; L': head to anus; mbd: max body diameter; sl: stoma length; sd: stoma diameter; pl: pharynx length; cl: corpus length; c/p: corpus length/pharynx length in %; mbbd: median bulb diameter; tbbd: terminal bulb diameter; ae-v: head to vulva; rl: rectum length; abd; anal body diameter; spic: spiculum length; gub: gubernaculum length; measurements are given in *μ*m; average in front of and minima/maxima in parenthesis.

### *Pristionchus marianneae *n. sp

Diagnostic character: The species is characterized by a 471 bp sequence of a 1 kb fragment of the small subunit ribosomal RNA gene (*SSU*) that was amplified by PCR with the primers shown in Materials and Methods. This *SSU *sequence is distinct from the *SSU *sequence of all other *Pristionchus *species, but is identical between all isolates of *P. marianneae*. The sequence is shown in Fig. 2.

GenBank accession code: DQ419901

A second diagnostic character is provided by mating experiments. *P. marianneae *males mate with females of other isolates but not with females/hermaphrodites of other species.

Morphological measurements: see Table 2

Type host and locality: On *Popilia japonica *(Coleoptera: Scarabaeidae) near Geneva, New York, United States of America.

Etymology: R. J. S and M. H. want to dedicate this species to their mothers.

Frozen strain number: RS5108

Holotype: One male permanent slide No: SMNK-NEMA-T0022 (Natural History Museum Karlsruhe, Germany)

Paratypes: One male and one female permanent slide No: SMNK-NEMA-T0023; One male and one female permanent slide No: SMNK-NEMA-T0024

### *Pristionchus pauli *n. sp

Diagnostic character: The species is characterized by a 471 bp sequence of a 1 kb fragment of the small subunit ribosomal RNA gene (*SSU*) that was amplified by PCR with the primers shown in Materials and Methods. This *SSU *sequence is distinct from the *SSU *sequence of all other *Pristionchus *species, but is identical between all isolates of *P. pauli*. The sequence is shown in Fig. 2.

GenBank accession code: DQ419900

A second diagnostic character is provided by mating experiments. *P. pauli *males mate with females of other isolates but not with females/hermaphrodites of other species.

Morphological measurements: see Table 2

Type host and locality: On *Lichnanthe vulpina *(Coleoptera: Scarabaeidae) in Carver, Massachusetts, United States of America

Etymology: M. H. wants to dedicate this species to Dr. Paul S. Robbins, an outstanding beetle expert and true friend.

Frozen strain number: RS5130

Holotype: One male permanent slide No: SMNK-NEMA-T0025 (Natural History Museum Karlsruhe, Germany)

Paratypes: One male and one female permanent slide No: SMNK-NEMA-T0026; One male and one female permanent slide No: SMNK-NEMA-T0027

### *Pristionchus americanus *n. sp

Diagnostic character: The species is characterized by a 471 bp sequence of a 1 kb fragment of the small subunit ribosomal RNA gene (*SSU*) that was amplified by PCR with the primers shown in Materials and Methods. This *SSU *sequence is distinct from the *SSU *sequence of all other *Pristionchus *species. The sequence is shown in Fig. 2.

GenBank accession code: DQ419904

*P. americanus *males do not mate successfully with females/hermaphrodites of other species.

Morphological measurements: see Table 2

Type host and locality: On *Polyphylla fullo *(Coleoptera: Scarabaeidae) in Centerville, Massachusetts, United States of America

Etymology: This species has been found only in the United States of America so far. Frozen strain number: RS5140

Holotype: One male permanent slide No: SMNK-NEMA-T0019 (Natural History Museum Karlsruhe, Germany)

Paratypes: One male and one female permanent slide No: SMNK-NEMA-T0020; One male and one female permanent slide No: SMNK-NEMA-T0021

### *Pristionchus pseudaerivorus *n. sp

Diagnostic character: The species is characterized by a 472 bp sequence of a 1 kb fragment of the small subunit ribosomal RNA gene (*SSU*) that was amplified by PCR with the primers shown in Materials and Methods. This *SSU *sequence is distinct from the *SSU *sequence of all other *Pristionchus *species, but is identical between all isolates of *P. pseudaerivorus*. The sequence is shown in Fig. 2.

GenBank accession code: DQ419902

Morphological measurements: see Table 2

Type host and locality: On *Phyllophaga sp. *(Coleoptera: Scarabaeidae) near Lincoln, Nebraska, United States of America.

Etymology: The species is very similar in respect to morphological measurements and molecular sequence to *P. aerivorus*. Mating experiments revealed that crosses between *P. pseudaerivorus *and *P. aerivorus *can result in sterile females.

Frozen strain number: RS5139

Holotype: One male permanent slide No: SMNK-NEMA-T0028 (Natural History Museum Karlsruhe, Germany)

Paratypes: One male and one female permanent slide No: SMNK-NEMA-T0029; One male and one female permanent slide No: SMNK-NEMA-T0030

### *Pristionchus *species sampled on American scarab beetles are predominantly gonochoristic

**Figure 3:**
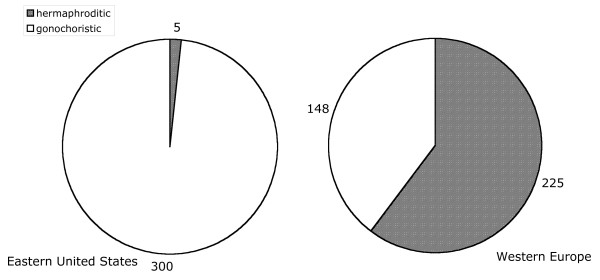
Pie charts indicating the ratio of gonochoristic (white) to hermaphroditic (grey) *Pristionchus *isolates in the Eastern US and Western Europe.

The result of the US survey indicates a striking difference in the mode of reproduction of North American and European beetle associated *Pristionchus *species. *P. aerivorus*, *P. pseudaerivorus *n. sp., *P. pauli *n. sp., *P. marianneae *n. sp. and *P. americanus *n. sp. are gonochoristic (male/female) species and account for 98% of the isolates from scarab beetles (Fig. 3). In contrast, more than 60% of the isolates from scarab beetles in Western Europe belong to two hermaphroditic species, *P. maupasi *and *P. entomophagus*, respectively (Fig. 3) [8]. Thus, North America and Europe differ in the *Pristionchus *species associated with scarab beetles and in the mode of reproduction of these nematodes.

### The *Pristionchus *species from the US form a phylogenetic clade together with *P. maupasi*

**Figure 4:**
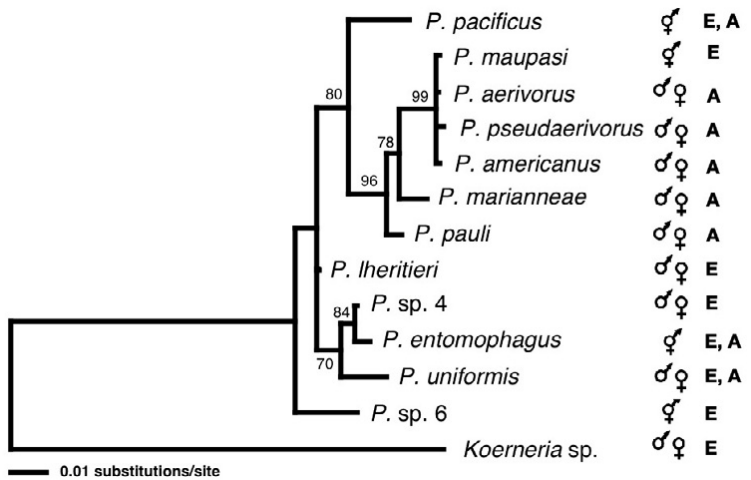
**Phylogenic maximum likelihood tree based on *SSU *sequences of the genus *Pristionchus***. As outgroup the sequence of *Koerneria *sp. was included as the closest related genus to *Pristionchus*. A single best tree was obtained using the Kimura 2-parameter model (Kimura 1980) with Ts/Tv = 1,4727, equal base frequencies, and a γ-correction with shape parameter α = 0,2381. Numbers at nodes indicate bootstrap values after 1000 replications. E, species observed in Western Europe; A, species observed in North-America (this study).

**Figure 5:**
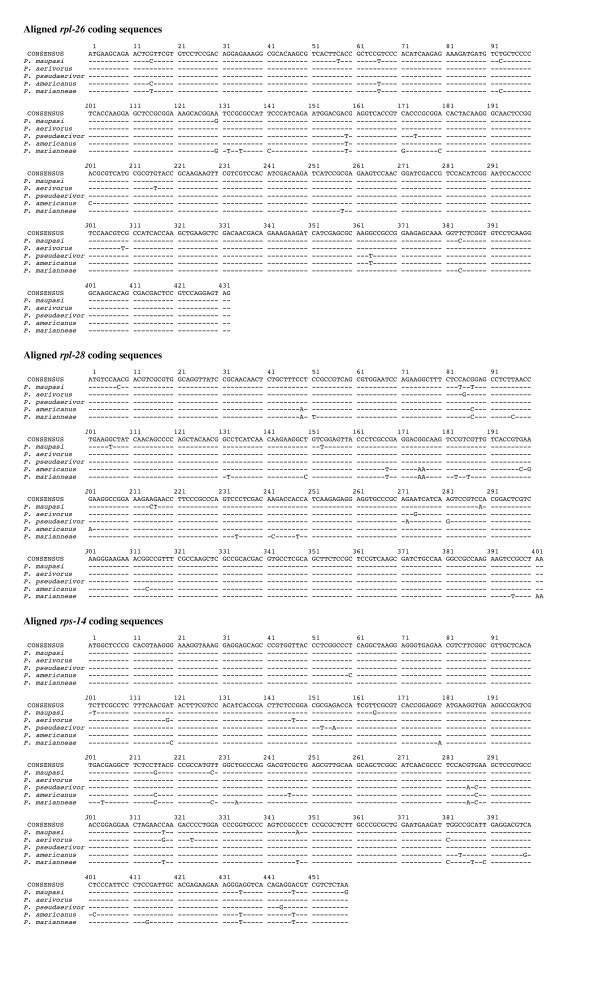
**Alignment of the coding sequences of the ribosomal protein genes *rpl-26*, *rpl-28*, and *rps-14 *of *P. maupasi*, *P. aerivorus*, *P. pseudaerivorus*, *P. americanus*, and *P. marianneae***. Dashes (-) indicate identity to the majority consensus sequence at the top.

To determine if the *Pristionchus *biogeography would match species phylogeny we incorporated the North American species into the phylogenetic framework of the genus *Pristionchus *with a representative of the closely related genus *Koerneria *as outgroup (Fig. 4). Interestingly, *P. aerivorus*, *P. pseudaerivorus *n. sp., *P. pauli *n. sp., *P. marianneae *n. sp. and *P. americanus *n. sp. form a single clade and are much more closely related to one another than the species found in Europe. *P. aerivorus, P. pseudaerivorus *n. sp. and *P. americanus *n. sp. are the most closely related species and differ in only one out of 471 base pairs in their *SSU *sequence (Fig. 2). Surprisingly, the cockchafer-associated hermaphroditic species *P. maupasi *from Europe belongs to the same clade and carries an *SSU *sequence identical to the *P. aerivorus *sequence (Figs. 2, 4). A more detailed analysis including several ribosomal protein encoding nuclear genes distinguishes the two species and indicates that *P. aerivorus *and *P. pseudaerivorus *n. sp. are the two most closely related species and that *P. maupasi *is the sister taxon to the *P. aerivorus/P. pseudaerivorus *n. sp. species pair (Fig. 5). Specifically, *P. aerivorus *and *P. pseudaerivorus *n. sp. differ in 19 nucleotides of the concatenated sequences of the *rpl-26*, *rpl-28 *and *rps-14 *genes. In contrast, *P. maupasi *and *P. aerivorus *differ in 31 nucleotides and *P. maupasi *and *P. pseudaerivorus *n. sp. differ in 33 nucleotides of the corresponding sequences (Fig. 5). *P. americanus *n. sp. and *P. marianneae *n. sp. show even more nucleotide differences to the other species (Fig. 5). Thus, the investigated genes allow us to clearly distinguish these four species.

### *P. aerivorus *and *P. pseudaerivorus *n. sp. follow Haldane's rule

**Figure 6:**
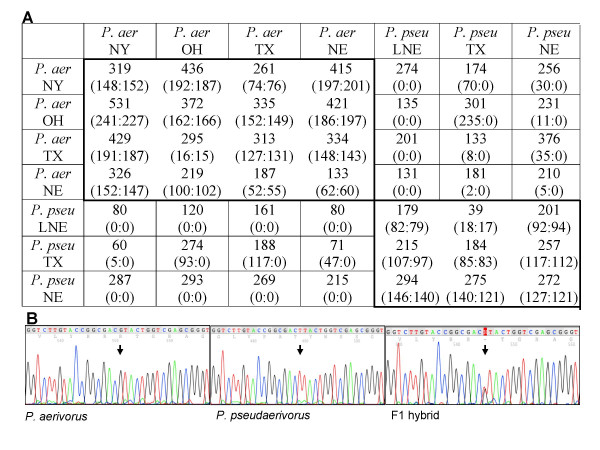
**F1 hybrids of P. *aerivorus *and *P. pseudaerivorus *n. sp. follow Haldane's rule**. (**A**) Results of crosses between four strains of *P. aerivorus (P. aer) *from New York (NY), Ohio (OH), Texas (TX) and Nebraska (NE) and three strains of *P. pseudaerivorus n. sp. (P. pseu) *from Texas (TX), Indian Cave, Nebraska (NE) and Lincoln, Nebraska (LNE). Each strain was crossed with all other strains in a reciprocal manner. Columns represent female animals and rows represent males used for crosses. Mating was performed in triplicate, placing one virgin female and one male on a plate. Numbers show the total of eggs laid by one female. In brackets, the number of eggs that developed into phenotypic female vs. male progeny is indicated. The difference between the upper number and the sum of the numbers in brackets is accounted for by unfertilized eggs and embryonic lethality commonly observed after crosses within and between animals of these two species. The given numbers represent the result of the experiment with the highest number of offspring. (**B**) *SSU *sequence profile of *P. aerivorus*, *P. pseudaerivorus *n. sp. and a hybrid between the two species.

The unusually high DNA sequence similarity between *P. aerivorus *and *P. pseudaerivorus *n. sp. suggests that they result from a recent speciation event. Therefore, these two species might still possess the potential to mate and form hybrid offspring. To obtain support for this hypothesis, we carried out cross-species mating experiments and found a strong case of Haldane's rule (Fig. 6). Haldane's rule states that in hybrids between diverging species the sterile absent or underrepresented offspring is of the heterogametic sex [11]. We found F1 hybrids in 12 of 24 crosses between *P. aerivorus *and *P. pseudaerivorus *n. sp. and all of the F1 hybrid animals were phenotypically female (Fig. 6A). Specifically, F1 hybrids were obtained after reciprocal crosses of P. *pseudaerivorus *n. sp. from Texas with four strains of *P. aerivorus*. In contrast, *P. pseudaerivorus *n. sp. from Nebraska produced F1 hybrids only when males were used for mating with *P. aerivorus *females. Another *P. pseudaerivorus *n. sp. strain from Lincoln, Nebraska (LNE) did not produce F1 hybrids with any of the *P. aerivorus *strains. Thus, the occurrence of species hybrids is highly strain-specific and does not correlate with the geographic origin of the strains used in the analysis.

The primary signal in *P. pacificus *and *C. elegans *sex determination is the ratio of the set of X chromosomes to autosomes [1]. Animals with two X chromosomes develop as females (XX), whereas animals with a single X chromosome develop as males (XO). Theoretically, the absence of males among the F1 hybrid animals could result from the death of the putative XO animals or the sexual transformation of these animals into females. The latter scenario was observed in crosses of *Caenorhabditis remanei *with *Caenorhabditis briggsae *[16].

Additional support for the results of the laboratory crosses came from genotyped *Pristionchus *nematodes from wild scarab beetles. We found two cases of hybrids between *P. aerivorus *and *P. pseudaerivorus *n. sp. emerging from individual beetles (Fig. 6B). Genotyping of other *Pristionchus *individuals from the same two beetles revealed that they carried both *P. aerivorus *and *P. pseudaerivorus *n. sp., suggesting that the observed hybrids are the result of cross-species fertilization (Fig. 6B). However, both hybrid animals were isolated more than a week after the beetle was placed on the petri dish. Therefore, it remains unknown if the observed hybrids came from the generation that lived on the beetle or whether it originated from fertilization in the laboratory. Taken together, *P. aerivorus *and *P. pseudaerivorus *n. sp. co-exist on the same beetles in nature, they can form hybrids and laboratory-derived F1 hybrids follow Haldane's rule. In addition, both species have a similar geographic distribution in our sampling.

### The Colorado potato beetle hosts the same *Pristionchus *species in North America and Europe

Finally, we wanted to know if *Pristionchus *nematodes occur in association with the CPB *Leptinotarsa decemlineata *in the United States. Our analysis of CPB in Europe had indicated that one species, *P. uniformis*, infests these beetles with high specificity [8]. The CPB is a pest species that originated in Mexico, where it extended its range of host plants from the wild solanaceae buffalo bur (*Solanum rostratum*) to include the potato (*Solanum tuberosum*). It migrated further north to the United States and was first described in Colorado in 1811. Spreading towards the East it reached the Atlantic coast in 1874 and was first reported in Europe in 1877 [17,18].

We collected CPB from two different locations in Ohio and New York State and found *Pristionchus *nematodes with an infestation rate of up to 60% (Table 1). Sequence analysis and mating experiments revealed that all of these isolates belong to *P. uniformis*, the same species that is found on European CPB. Thus, in contrast to scarab beetles, the CPB hosts the same (and only one) *Pristionchus *species on both continents, *P. uniformis*. Given the recent invasion of the CPB to Europe we speculate that *P. uniformis *was introduced to Europe together with its beetle vector, the CPB, at the end of the 19th century.

## Discussion

We have shown that the *Pristionchus *species associated with scarab beetles in the Eastern United States are different from those found in Western Europe. The four predominant American species *P. aerivorus*, *P. pseudaerivorus *n. sp., *P. marianneae *n. sp. and *P. pauli *n. sp. are all unknown from Western Europe. In general, there is some preference of certain *Pristionchus *nematodes for a specific beetle. However, the nematode – beetle association is not striktly species-specific, an observation that is similar to what has been observed in our study in Europe [8]. This very interesting and important issue awaits further analysis, including more comprehensive studies on additional continents and exhaustive samplings of one particular continent.

One surprising observation of our surveys in the United States and Europe is the difference in the mode of reproduction observed between the two continents. While the two predominant *Pristionchus *species on European scarab beetles are hermaphroditic, the four predominant American species, representing 98% of the American isolates, are gonochoristic. We speculate that the evolution of hermaphroditism is a chance event that occurs with a very low probability. Despite this fact, the *Pristionchus *phylogeny presented in this study strongly suggests that the evolution of hermaphroditism has occurred multiple times independently: The hermaphroditic species *P. pacificus*, *P. maupasi *and *P. entomophagus *belong to three different clades of the genus and all of them have gonochoristic sister species (Fig. 4). Two different explanations can account for this pattern. First, speciation of hermaphroditic species might be absent or at least not very frequent. Alternativley, more frequent extinctions of hermaphroditic lineages than gonochoristic ones can also explain the observed patterns. The latter hypothesis is further supported by the fact that hermaphroditism is mostly observed in terminal lineages. Similar results have been obtained for rhabditid species of the genus *Caenorhabditis *[19].

The satellite organism *P. pacificus *represents an independent clade in the *Pristionchus *phylogeny with only three isolates from scarab beetles from Ohio, Nebraska and Massachusetts. *P. pacificus *does not seem to be a common species on scarab beetles in the United States, a finding that raises two possibilities. First, *P. pacificus *occurs in the United States, but not in association with the beetles that were investigated in this study. Second, *P. pacificus *represents a species that has invaded North America recently and is not yet very common.

Several observations provide a first hint that the North American *Pristionchus *species are the result of relatively recent speciation events. The four most common beetle-associated species form a single clade in the phylogenetic tree. The only European species in this clade is *P. maupasi*, indicating that there is no complete biogeographic separation. Furthermore, this observation suggests that *P. maupasi *derives from a lineage that dispersed to Europe from North America. *P. aerivorus *and *P. pseudaerivorus *n. sp. co-exist on the same beetle and cross-species matings were observed under laboratory conditions following Haldane's rule. All F1 hybrid animals were sterile females. We speculate that the effect of recessive deleterious alleles in the heterogametic hybrids causes inviability of males, a phenomenon known as dominance theory [20]. Given the easiness with which *Pristionchus *strains can be obtained at various places in the United States, *Pristionchus *nematodes provide an interesting model system to study if sympatric speciation occurs in nematodes and to study speciation and hybridization processes in general.

Finally, our studies of the CPB in the United States and Europe suggest a case of a species invasion. The introduction of the American CPB to Europe represents one of the first cases in which the invasion of an agricultural pest species was well documented. Recent studies have revealed that the genetic diversity of the CPB is much higher in North America than in Europe [21]. This observation is consistent with the assumption that the original invasion was due to a small number or even a single founder event. The fact that the CPB in the United States and Europe hosts the same nematode, *P. uniformis*, strongly suggests that *P. uniformis *was introduced into Europe with its beetle vector. With more *P. uniformis *isolates in hand from both continents we can start to address the question whether the genetic variability of European *P. uniformis *populations is reduced when compared to that of American populations. More generally, such studies can provide an important model for nematode species invasions across continents. In this context it is important to note that the case of *Pristionchus *is not unique for nematode invasions by insect vectors. One recent case is of high commercial interest. The pine wood nematode *Bursaphelenchus xylophilus *is a major pathogen of conifers and has recently been introduced to Europe by its vector, the beetle *Monochamus galloprovincialis *[22].

In summary, the observations of the *Pristionchus *species pattern on scarab beetles and the CPB, as well as the easiness and frequency with which these nematodes can be isolated, indicates the potential of this nematode group for studying speciation, biodiversity, biogeography and species invasion on a global scale. When future samplings provide a global pattern of *Pristionchus *species and associations, the combination of field studies with genetic manipulation under laboratory conditions will allow functional investigations into nematode biodiversity and biogeography.

## Conclusion

We describe the *Pristionchus *– beetle association in the Eastern United States and show striking evolutionary and ecological patterns. Most *Pristionchus *isolates from American scarab beetles belong to five species, all of which differ from the European species. The two most closely related species, *P. aerivorus *and *P. pseudaerivorus *n. sp., follow Haldane's rule. The Colorado potato beetle is associated with the same species, *P. uniformis *in the United States and Europe, suggesting that *P. uniformis *was introduced to Europe together with its beetle vector. Together, the *Pristionchus *– beetle association provides a powerful tool for studying speciation, biodiversity, biogeography and species invasion on a global scale.

## Materials and methods

### Isolation of nematodes

We collected different beetles at adult stage using sweeping nets, blacklight traps and pitfall traps baited with dung. The beetles were transferred to the lab alive, sacrificed by cutting them in half transversely, and put on NGM agar plates (6 cm diameter) seeded with 300 *μ*l of the slowly growing *E. coli *strain OP50. The plates were checked daily using a Zeiss Stemi 2000 dissecting scope over a period of one to three weeks for emerging and reproducing nematodes. From the emerging nematodes we produced isogenic lines by transferring single gravid females or hermaphrodites to new plates. To see whether the isolated nematodes belonged to gonochoristic or hermaphroditic species, virgin larvae were singled out onto plates. The presence of offspring indicated that they represent a hermaphroditic species.

Emerging nematodes were determined to family level with a Zeiss Stemi 2000 dissecting scope and to genus level with a Zeiss Axioplan 2 microscope using the key by Sudhaus and Fürst von Lieven [9]. For determination several worms were transferred onto microscopic slides covered with a 0.5 mm thick layer of 5% agar and either immobilized by heating the slide over an open flame to about 60°C for a few seconds or anesthesized with sodium azide. Within the genus *Pristionchus *many species can not be identified by morphological methods. We therefore chose to use molecular tools and mating experiments with reference strains to distinguish the different species.

We have originally isolated up to three isogenic female lines per beetle and have processed them for sequence analysis. In nearly all cases, these isogenic female lines had identical *SSU *sequences. The exceptions were the observed hybrids between *P. aerivorus *and *P. pseudaerivorus *n. sp. described above, and two cases where isogenic female lines of *P. aerivorus *and *P. pseudaerivorus *or *P. aerivorus *and *P. marianneae *were derived from the same beetle. When the *SSU *sequences of different isogenic female lines from the same beetle were identical, only one isolate was further considered. Throughout the text (and in the Tables and Figures), we consider only one of these identical isolates per beetle. In particular, the numbers and frequencies provided in Table 1 and Figure 3 consider only one of these isolates. In Table 1, beetle individuals from the different sampling sites are pooled. There was little variation in incidence of *Pristionchus *species on beetles among sampling sites.

### Molecular species identification

Molecular species identification was done using the small subunit rRNA gene (*SSU*) as described before [8]. In short, genomic DNA from single nematodes was prepared using the NaOH digestion procedure described by Floyd et al. [23]. A single worm was transferred to 20 *μ*l of 0.25 M NaOH, incubated overnight at 25°C and heated to 99°C for 3 min before 4 *μ*l of 1M HC1, 10 *μ*l of 0.5 M Tris-HCl (pH 8.0) and 5 *μ*l of 2% Triton X-100 were added. The mixture was heated to 99°C for 3 min, frozen at -20°C and reheated at 99°C for further 3 min. Two microliters of this extract were used for subsequent polymerase chain reaction (PCR).

A 1 kb fragment of the *SSU *was amplified by PCR using the primers SSU18A (5'-AAAGATTAAGCCATGCATG-3') and SSU26R (5'- CATTCTTGGCAAATGCTTTCG-3') [23,24]. The reactions were performed in 25 *μ*l of 1× PCR buffer (Amersham Biosciences, Freiburg, Germany) containing 2.5 mM of MgCl_2_, 0.16 mM of each deoxynucleoside triphosphate, 0.5 *μ*M of each primer, 2 *μ*l of the lysate, and 2 units of *Taq *DNA polymerase (Amersham). The reactions were started by initial denaturation at 95°C for 2 min in a PTC-200 (MJ Research, Biozym, Hess. Oldendorf, Germany) or T gradient (Biometra, Göttingen, Germany) thermocycler, followed by 35 to 40 cycles of denaturation at 95°C for 15 sec, primer annealing at 50°C for 15 sec, and extension at 72°C for 2 min. A final incubation step at 72°C for 7 min concluded the reaction. PCR products were purified by the Qiagen PCR product gel extraction kit (Qiagen, Hilden, Germany). Approximately 500 bp of the 5'-terminal end were sequenced using the primer SSU9R (5'-AGCTGGAATTACCGCGGCTG-3') and the Big Dye terminator protocol (Applied Biosystems, Darmstadt, Germany).

Sequences were aligned manually using the Seqpup 0.6f software for Macintosh [25]. The substitution model for the reconstruction of phylogenetic relationships was selected by the hierarchical likelihood ratio test as implemented in the Modeltest 3.7 software [26]. The selected substitution model corresponds to the Kimura 2-parameter model [27] with Ts/Tv = 1.4727, equal base frequencies, and a γ-correction with shape parameter α = 0.2381.

### Phylogenetic analysis

Phylogenetic trees were determined using the heuristic search algorithm under the maximum likehood (ML) criterion using PAUP*4.0bl0 program [28]. Trees were rooted by the *Koerneria *sp. sequence as outgroup. Neighbour joining (NJ, [29]) and maximum parsimony (MP) trees were drawn by the same program. Alignment gaps were eliminated from the analysis. The topological stability of the trees was assessed by 1000 bootstrap replications [30].

### Mating experiments

To confirm the species identification by the molecular sequence of a novel isolate we performed mating experiments with the reference strain of the respective species (see below for definitions). Five virgin females were put on a plate with a small spot of OP50 together with five males of the reference strain of a certain species. On a second plate we picked the opposite sexes of the two strains to test for reciprocity. If there was no offspring after one week, the experiments were repeated two more times. If fertile offspring occurred we considered the two strains to belong to the same species.

### Isolate and strain definitions

We use the following definitions to distinguish "isolates" and "strains". An isolate is an isogenic female line, which is derived from a beetle sample and subjected to molecular and experimental analysis. After species identification we established one isolate per species and location as a strain. The strains are permanently cultured in the lab, have a strain number and are also kept as frozen stocks. For each new species designated by molecular sequence analysis and mating experiments, one strain was defined as a reference strain (see below).

### Assigning names to reference strains

According to the designation of reference strains, the 285 isolates obtained from American scarab beetle material fell into seven *Pristionchus *species, two of which were already cultured in our lab (*P. pacificus*, reference strain PS312 and *P. entomophagus *ref. strain RS0144). The other five could not be identified. We found the morphometric data of the most common of the unidentified species to coincide with the description of the only North American *Pristionchus *species listed in the catalog provided by Sudhaus and Fürst von Lieven [9], namely *P. aerivorus*. The other four species are novel and are described based on morphometric analysis, *SSU *sequence analysis and mating experiments (Fig. 2, Table 2 and species diagnoses). In total, the following eight *Pristionchus *species were obtained from scarab beetles (seven species) and the Colorado potato beetle (one species) in North America:

*Pristionchus entomophagus *(Steiner, 1929) (ref. str. RS0144)

*Pristionchus pacificus *Sommer, Carta, Kim & Sternberg, 1996 (ref. str. PS312)

*Pristionchus uniformis *Fedorko & Stanuszek, 1971 (ref. str. RS0141)

*Pristionchus aerivorus *(Cobb in Merrill & Ford, 1916) (ref. str. RS5106)

*Pristionchus pseudaerivorus *n. sp. Herrmann, Mayer & Sommer 2006 (ref. str. RS5139)

*Pristionchus marianneae *n. sp. Herrmann, Mayer & Sommer 2006 (ref. str. RS5108)

*Pristionchus pauli *n. sp. Herrmann, Mayer & Sommer 2006 (ref. str. RS5130)

*Pristionchus americanus *n. sp. Herrmann, Mayer & Sommer 2006 (ref. str. RS5140)

### Comparison of ribosomal proteins

In order to obtain nuclear protein coding genes to distinguish *P. americanus *n. sp. from its sister species *P. aerivorus*, *P. pseudaerivorus *n. sp. and *P. maupasi *we isolated total RNA from 50 to 100 *μ*g of nematodes using the TRIZOL reagent (Invitrogen). The RNA was reverse transcribed into cDNA with the help of the Omniscript reverse transcriptase kit (Qiagen, Hilden, Germany) and the primer RH5620 (5'-GAAGATCTAGAGCGGCCGCCCTTTTTTTTTTTTTTT-3'). Based on EST data from SL1-transspliced genes from European *Pristionchus *species (unpublished data) we designed generic RT-PCR primers for *Pristionchus *ribosomal protein genes (WM8220, 5'-TCGACAACGACAGAAAGAAGA-3', *rpl-26*, sense; WM8221, 5'-ACGGAGTCRTCGCTGTRCTTGC-3', *rpl-26*, antisense; WM8263, 5'-CCGTCAGCGYGGMATCCAGAAG-3', *rpl-28*, sense; WM8264, 5'-GCTGGASGGAGCGGAGRAGCTG-3', *rpl-28*, antisense; WM8114, 5'-GCYCAYATYTTCGCYTCTTTCAA-3', *rps-14*, sense; WM8113, 5'-GGRGTCTTNGTTCTRGTTCCTC-3', *rps-14*, antisense) and used them to synthesize the complete transcript in two overlapping fragments by PCR. The SL1-specific primer BJ234 (5'-GGTTTAATTACCCAAGTTTGAG-3') was used with the antisense primers to obtain the 5' part of the transcripts and the combination of RH5620 and the sense primers to obtain the 3' parts. The PCR was performed with the help of the HotStar *Taq *DNA polymerase kit (Qiagen) including the Q solution in the reaction mix. Conditions were initial activation of the enzyme at 95°C for 15 min, followed by 40 cycles of denaturation at 94°C for 30 sec, primer annealing at 50°C for 30 sec, and primer extention at 72°C for 3 min. The reaction was completed by an incubation at 72°C for 10 min. PCR fragments were gel purified using the Wizard SV gel purification kit (Promega) and sequenced directly using the PCR primers.

## Authors' contributions

MH carried out all of the field work and the generation of isogenic female lines and crosses. WEM generated isogenic female lines and did all of the molecular analysis. RJS designed and discussed the experiments with MH and WEM and wrote, together with MH and WEM the manuscript.
